# Delayed Eruption of Permanent Dentition and Maxillary Contraction in Patients with Cleidocranial Dysplasia: Review and Report of a Family

**DOI:** 10.1155/2018/6591414

**Published:** 2018-07-04

**Authors:** A. Impellizzeri, G. Midulla, U. Romeo, C. La Monaca, E. Barbato, G. Galluccio

**Affiliations:** ^1^Department of Oral and Maxillofacial Sciences, Sapienza University of Rome, Rome, Italy; ^2^Private Practice, Rome, Italy; ^3^Private Practice, Montreux, Switzerland

## Abstract

**Introduction:**

Cleidocranial dysplasia (CCD) is an inherited disease caused by mutations in the RUNX2 gene on chromosome 6p21. This pathology, autosomal dominant or caused by a spontaneous genetic mutation, is present in one in one million individuals, with complete penetrance and widely variable expressivity.

**Aim:**

To identify the incidence of these clinical findings in the report of the literature by means of PubMed interface from 2002 to 2015, with the related keywords. The report of local patients presents a clinical example, related to the therapeutic approach.

**Results and Discussions:**

The PubMed research resulted in 122 articles. All the typical signs were reported in all presented cases. The maxilla was hypoplastic in 94% of the patients. Missing of permanent teeth was found in two cases: one case presented a class II jaw relationship, instead of class III malocclusion. Similar findings were present in our cohort.

**Conclusion:**

CCD is challenging for both the dental team and the patient. The treatment requires a multidisciplinary approach. Further studies are required to better understand the cause of this disease. According to this review, a multistep approach enhances the possibilities to achieve the recovery of the most possible number of teeth, as such to obtain a good occlusion and a better aesthetic.

## 1. Introduction

Cleidocranial dysplasia (CCD) was first described by Pierre Marie and Paul Sainton in 1898 as “dysostose cléido-crânienne héréditaire,” mutational dysostosis, or cleidocranial dysostosis. Though the first description of cleidocranial dysostosis is credited to Meckle in 1760, the combination of clavicular and cranial defects was identified by Scheuthauer in 1871.

CCD is an autosomal dominant inherited disease caused by mutations in the RUNX2 gene on chromosome 6p21 encoding a transcription factor involved in osteoblast differentiation and skeletal morphogenesis. This pathology is transmitted as an *autosomal dominant trait* or it is caused by a *spontaneous genetic mutation* and is present at a frequency of *one in one million individuals*. It presents *complete penetrance* and *widely variable expressivity*.

The term “cleidocranial dysostosis” has been used; however, the syndrome got renamed aptly from cleidocranial dysostosis to cleidocranial dysplasia after the conference for constitutional disorders of bone held in Paris in 1969, given that *RUNX2* has important functions both during skeletal formation and in bone maintenance, and CCD is affecting not only the clavicles and the skull but the entire skeleton [1, 2].

This rare disease is classified as #119600. A number sign (#) is used with this entry because of evidence that CCD is caused by heterozygous loss-of-function mutation in the RUNX2 gene (600211), encoding transcription factor CBFA1. Heterozygous duplication in RUNX2 resulting in a gain of function causes metaphyseal dysplasia and maxillary hypoplasia with or without brachydactyly (MDMHB; 156510). Pycnodysostosis (265800) and mandibuloacral dysplasia (248370) are disorders to be considered in the differential diagnosis of CCD. Acroosteolysis and bone sclerosis with tendency to fracture are differentiating features of pycnodysostosis.

Many case reports and case studies have been reported in the literature [3–7]. One of the most colorful families was described by Jackson [8]. The condition occurred in many descendants of a Chinese man named Arnold who embraced the Mohammedan religion and 7 wives in South Africa. Jackson in 1951 was able to trace 356 descendants, of whom 70 were affected by the “Arnold head” [8, 9].

Due to the wide variability, diagnosis solely based on the clinical features may be difficult. CCD is mainly characterized by a pathognomonic deformity of the skull (persistently open skull sutures with bulging calvaria), hypoplastic midface, lack of eruption of permanent teeth, supernumerary teeth, hypoplastic or aplastic clavicles, and multiple other malformations [10]. CCD makes also a special problem for the dentists due to a large number of abnormalities: highly arched palate, cleft palate, delayed union of mandibular symphysis, delayed tooth eruption, dental root and crown abnormalities, crypt formation around impacted teeth, and ectopic teeth. Dental abnormalities are sometimes a sole clinical sign of the mutation.

Early diagnosis of CCD is essential for introduction of an appropriate treatment approach based on interdisciplinary cooperation between orthodontists as well as oral and maxillofacial surgeons and general dentists at the right moment [11].

The main clinical features of CCD include persistently open skull sutures with bulging calvaria, hypoplasia, or aplasia of the clavicles permitting abnormal facility in opposing the shoulders, wide pubic symphysis, short middle phalanx of the fifth fingers, dental anomalies, and often vertebral malformation (Table 1).

Cooper et al. in 2001 assembled a series of 90 CCD individuals and 56 relative controls ascertained from genetic and dental practices in the United States, Canada, Europe, and Australia. A number of previously unrecognized complications were significantly increased, including scoliosis, pes planus, sinus infections, upper respiratory complications, recurrent otitis media, and hearing loss [12].

Dental abnormalities, including supernumerary teeth, failure of exfoliation of the primary dentition, and malocclusion, were found to be serious and complex problems that required intervention. The combination of normal deciduous teeth, delayed eruption of permanent teeth, and multiple impacted supernumerary teeth is practically diagnostic of CCD.

The increase in odontogenesis leads to excessive number of supernumerary teeth. A study by *Yamamota* et al. concluded that an early loss of gubernacular cords resulted in failure of a tooth to erupt [13].

As stated by Roberts et al. in 2013, “the supernumerary teeth in CCD may result from the lack of inhibition or incomplete resorption of tooth bud formation. Supernumerary teeth may also result from the presence of remnants of dental laminae following dental extraction. These epithelial cell rests are usually resorbed during the normal tooth morphogenesis” [9].

**Figure 1:**
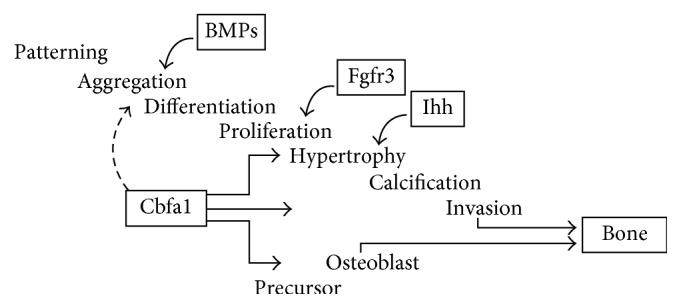
Role of Cbfa1 in bone formation (from [10]).

It has been argued that CBFA1 mutations segregate with the CCD phenotype and that heterozygous loss of function is sufficient to produce the characteristic clinical findings. The CBFA1, a subunit of the core binding factor (CBF) transcription factors, probably plays a significant role in bone development, as it has been revealed by mutated mice in which the *Cbfa1* was completely deficient and died immediately after birth owing to a complete absence of bone. The cause was identified in an arrest in endochondral as well as membranous bone formation. Moreover, the stimulation of cells that normally do not express *Cbfa1* with BMP7 (a secreted molecule that can induce bone formation) leads to expression of *Cbfa1* before the expression of any other osteoblast marker genes identifying *Cbfa1* as an inducer of osteoblast differentiation (Figure 1).

Very recent findings suggest that *Cbfa1* is not only essential for osteoblast formation, but also a major regulator of chondrocyte differentiation. In *Cbfa1* (−/−) mice chondrocytes, hypertrophy within this cartilaginous model does not take place, indicating a role for *Cbfa1.* In addition, also vascular invasion of calcified cartilage does not take place [12].

Throughout the years, different clinical approaches for the treatment of patients with CCD have been suggested in the literature [13, 14].

1. The Toronto-Melbourne approach is based on timed, serial extractions of deciduous teeth; it depends on the extent to which the roots of the permanent teeth have developed, so for this approach, the best period for treatment is in the initial stages with a combined oral surgery and orthodontic approach. It is recommended that surgical procedures should begin at about 5 to 6 years of age with extraction of the anterior deciduous teeth (Table 2). Later (6 or 7 years of age), the deciduous incisors are exposed, and healing is allowed; orthodontic brackets are placed on the permanent incisors as soon as possible, and the deciduous posterior teeth are extracted. When the patient is 9 or 10 years of age, the permanent premolars are exposed and allowed to heal. Thereafter, orthodontic brackets are placed on the permanent canines and premolars. During each procedure, which is performed under general anesthesia, the supernumerary teeth are also removed together with the bone covering the underlying permanent teeth.
2. The Belfast-Hamburg approach is a single surgical approach that limits the number of surgeries to a single episode. All deciduous and supernumerary teeth are extracted, and all unerupted permanent teeth are exposed simultaneously under general anesthesia. Surgical packing is placed, and healing occurs by secondary intention. The surgical packs are changed frequently until brackets can be bonded into place. Orthodontic appliances are placed on the few fully erupted teeth, and elastics are tied to the unerupted teeth to encourage eruption (Table 3).
3. The Jerusalem approach is based on at least 2 surgical interventions, depending on the root development of the permanent dentition. In the first phase, which begins approximately at 10 to 12 years of age, the anterior teeth are involved in the treatment in phase 2 (age 13 years and older), and the posterior teeth are treated. These stages are carried out simultaneously in both jaws under general anesthesia. One of the goals of this approach is to immediately deal with the absence of anterior teeth by placing an orthodontic appliance to erupt the anterior teeth first (Table 4).
4. The “Bronx Approach” uses an interim overdenture prosthesis during the long course of treatment [15]. The timing of the surgical intervention is dependent on the root development of the permanent teeth (Table 5).

### 1.1. Aim of the Study

The aim of the study is to identify the incidence of delayed eruption of permanent teeth and other dentoalveolar findings, that is, supernumerary teeth, eruption failure, and hypoplastic upper jaw, on all clinical reports found on international literature by means of PubMed interface form January 2002 to December 2015. We investigated the incidence of high-arch palate and contraction of the upper jaw and the response to dentoalveolar or skeletal expansion using orthodontic appliances, such as TPA, Quad-helix, or REP. A local patient cohort with a family report of three patients has been analyzed, identifying most suitable treatment strategy.

## 2. Materials and Methods

The study is divided into two sections: a systematic review of the literature and the description of a family of two patients and their mother with CCD observed in our Department of Orthodontics of “Sapienza”-University of Roma.

### 2.1. Literature Review (LR)

#### 2.1.1. Inclusion and Exclusion Criteria

All case series and case reports on humans reporting description of clinical parameters in CCD-affected patients were included from January 2002 until December 2015. Grey literature was excluded. Only reports written in English, German, or French were included.

Papers that were primarily concerned with specific pathological findings or radiological modalities were not included. All reports that contained only genotype descriptions were excluded. Studies about prenatal diagnosis of CCD were not included in the LR.

#### 2.1.2. Search Strategy

The PubMed interface of Medline (http://www.ncbi. nlm.nih.gov) was searched using the following key words: “Cleidocranial dysplasia,” “Dysostose cléido-crânienne,” “Cleidocranial dysostosis,” and “Osteo-dental dysplasia.”

The cases presented include literature reference, gender distribution, and patients' age at the time of presentation. Signs and symptoms were divided into the categories “supernumerary teeth,” “failure of tooth eruption,' “delayed eruption of permanent teeth,” “hypoplastic maxilla,” “transversal contraction of the maxilla,” “clavicular sign,” and “other skeletal disorders.” As described in the literature, the “clavicular sign” was defined by the patient's ability to oppose the shoulders in front as a result of hypoplastic or aplastic clavicles. The family history was also considered.

Furthermore, the aim was to investigate the answer of these patients to an orthopedic therapy using the rapid palate expander (REP), hypothesizing that most patients do not have a positive response to the device, needing surgical-assisted rapid palate expansion (SARPE) to correct the transversal discrepancies.

#### 2.1.3. Local Patient Cohort

The clinical features of two siblings and their mother, all affected by CCD, who came to our department of Orthodontics at Sapienza University of Rome are described. The patients came to our observation after being treated by a general dentist without any successful therapy. Ortopanoramic and lateral cephalometric radiographs were prescribed as well as a TC cone-beam to evaluate dental and skeletal conditions. Moreover, a detailed anamnesis was collected to evidence the pedigree of the family. All medical files were also considered to picture a precise diagnosis of the two patients, including the growth stage and prediction to elaborate an accurate treatment planning.

This study was approved by the Department of Oral and Maxillofacial Sciences—Umberto I Hospital IRB, and all participants signed an informed consent agreement.

## 3. Results

### 3.1. Genetics of CCD

Runx2 is part of a transcription factor complex that directs the differentiation of mesenchymal precursor cells toward mature osteoblasts. Mutations of R225 are most frequently identified with many independent CCD patients reported to date. R225 resides within a scratch of basic amino acids at the carboxy terminus of the runt domain. This motif has been shown to act as a nuclear localization signal, and mutations affecting R225 inhibit the nuclear accumulation of Runx2 protein [14, 16].

Runx2 is expressed in an initial period of embryonic development; it plays a significant role in both chondrocyte and osteoblast lineages. Osteoblasts take part in new bone formation.

*RUNX2* plays a significant role in the epithelial-mesenchymal interactions that control progressive tooth morphogenesis and histodifferentiation of the epithelial enamel organ.

## 4. Literature Review

The PubMed research resulted in 122 articles containing the keywords “Cleidocranial dysplasia,” “Dysostose cléido-crânienne,” “Cleidocranial dysostosis,” and “Osteo-dental dysplasia.” Among these 122 articles, 42 were excluded from the SR only by reading the title and the abstract, as they treated the following:

- Prenatal diagnosis of CCD (6 articles)
- Neonates with CCD (4 articles)
- Nonsyndromic conditions of patients presenting supernumerary teeth (8 articles)
- Related syndromes presenting similar symptoms as CCD such as Yunis-Varón Syndrome OMIM #216340 (6 articles), Ehlers Danlos syndrome OMIM #130000, Pycnodysostosis OMIM #265800 (2 articles), anophthalmia syndrome OMIM #206900 (1 article) other genetic mutations (4 articles)
- A cover picture was excluded from the LR
- A quiz was also excluded from the LR
- No full text available (9 papers).

After careful analysis of the remaining 80 full texts, it was possible to exclude further 23 papers for several reasons.  Table 6 shows the articles excluded and the rationale for exclusion.

**Figure 2:**
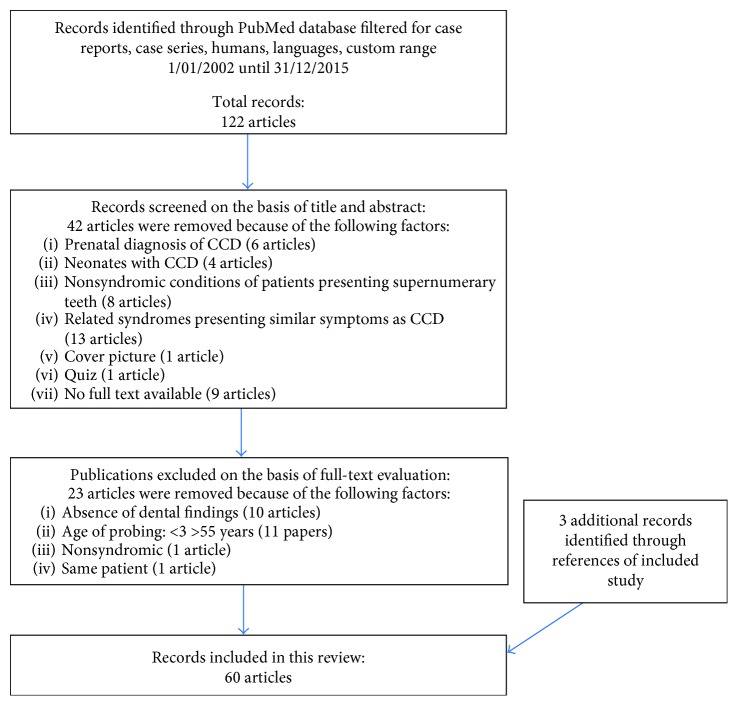
Results.

Three papers were specifically researched on the PubMed interface and included in the LR as perfectly matching the inclusion criteria. Finally, 60 were included in the SR as they reported the variables of interest (Table 7) (Figure 2).

Of all the variables reported, we analyzed those relative to the clinical signs of the syndrome and those related to the kind of treatment performed.

Among all the case reports analyzed, we can subdivide our population in two groups on the basis of gender (Table 8). The first group consists of 46 female patients with a mean age of 18.85 years. Supernumerary teeth were reported in all presented cases. There was an eruption failure and delayed eruption of permanent teeth in all cases as well. The maxilla was hypoplastic in 94% of the patients. Maxillary contraction and the clavicular sign were found in all the cases reported. Missing of permanent teeth was found in two cases: tooth 3.2 in one case and tooth 3.7 in the other case were missing.

Four patients were treated with orthognathic surgery for maxillary advancement. The contraction of the upper jaw was treated with different orthodontic appliances: REP, Hyrax, transpalatal arch (TPA), and removable appliance (Schwartz appliance) followed by Delaire mask. All answered positively to orthodontic therapy (Table 9).

The second group consists of 33 males, mean age 18 years. All presented supernumerary teeth, eruption failure, delayed eruption of permanent teeth, maxillary contraction, and the clavicular sign. The maxillary contraction was treated in one case with crisscross elastics and in two cases with a TPA. Of these patients, 94% had hypoplastic maxilla; one case presented a class II jaw relationship, whereas all the others had a class III malocclusion. Two patients underwent orthognathic surgery for maxillary advancement and mandibular setback (Table 10).

### 4.1. Family Report

A family of three subjects, a mother and two siblings, has been analyzed in all the details of the clinical findings. A complete examination, both for the oral characteristics and for the general relevant data, is reported. For each case, a possible treatment plane has been hypothesized following the three therapeutic approachs illustrated above.

#### 4.1.1. Case 1

A 12-year-old boy, S. R., came to an evaluation in October 2015 in the Orthodontic Department of Sapienza University of Rome. His weight was 24 kg (<3 centile) and height was 1.20 m (<3 centile), born from cesarean delivery at 39 weeks to a mother affected by CCD. He presented open fontanels and patent sutures at birth. Closure of anterior fontanel occurred at 3 years of age. In 2016, he underwent a complete clinical and auxological evaluation at the Department of Rare diseases of Sapienza University of Rome. His weight was 21.5 kg (25 centile) and height was 118.5 cm (25 centile), and the hand-wrist X-ray showed skeletal age of 6 years. Clinical examination showed narrow clavicles and accentuated joint mobility. The patient can oppose the shoulders on the midline. Orthopedic assessment showed left lumbar scoliosis and right dorsal scoliosis.

Audiological examination: normal audiometry and impedentiometry with tympanogram type A on the right and type C on the left. The cocleostapedial reflex was present bilaterally for both ipsilateral and contralateral stimulations.

*(1)* Laboratory findings: deficit of vitamin D 25-OH (17.5 ng/ml), Beta-Cross Laps levels of 0.81 ng/ml. DEXA *z*-score -2.8.

X-Ray of the spine (2 projections) and long bones (2 projections) shows pseudarthrosis of the medial third of both clavicles, that appears hypoplastic. Iliac wings appear squared and narrow. Hypoplastic pubic bones. Valgus femoral necks. Widened cephalic nuclei of the femoral bone. Proximal pseudoepiphysis of the second and fifth metacarpi in both hands. Brachy-telefalangy with hypoplastic nails. Retarded skeletal age between 6 and 7 years. TC of the head: mastoid appear thickened and ivory. Narrow antral cavities with thickened walls. Tympanic cavities with thickened walls. Cochlea surrounded by compact dense bone. Hyperemia of presphenoidal adenolymphoid tissues. Deviation of nasal septum.

**Figure 3:**
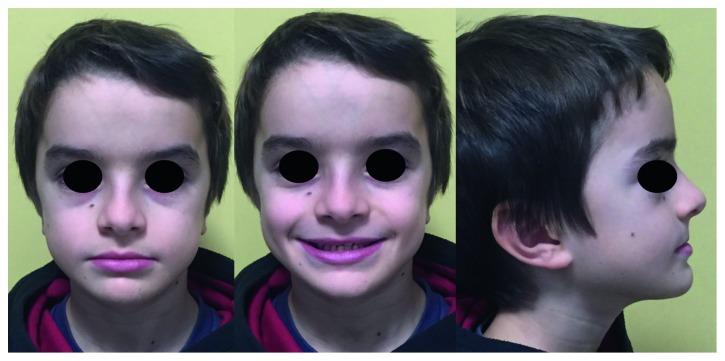
Case 1: pretreatment facial photographs.

**Figure 4:**
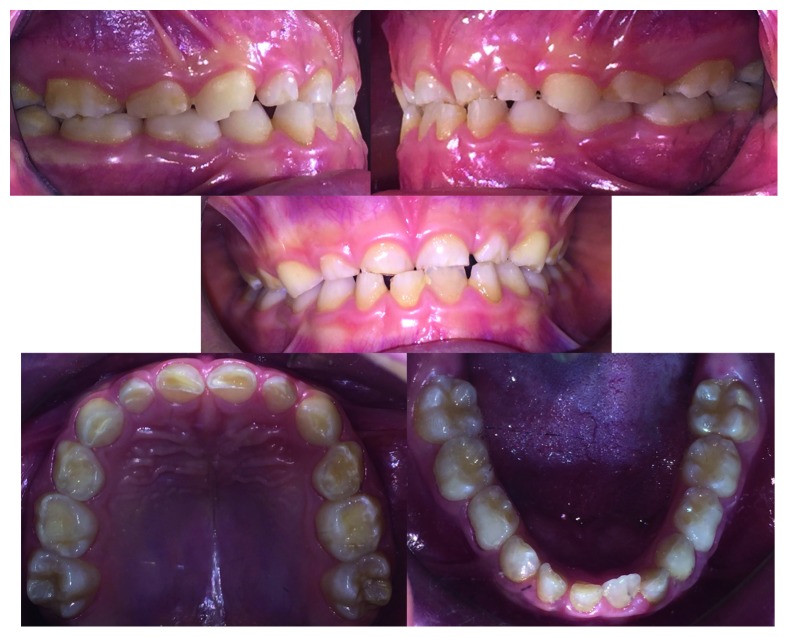
Case 1: intraoral photographs.

**Figure 5:**
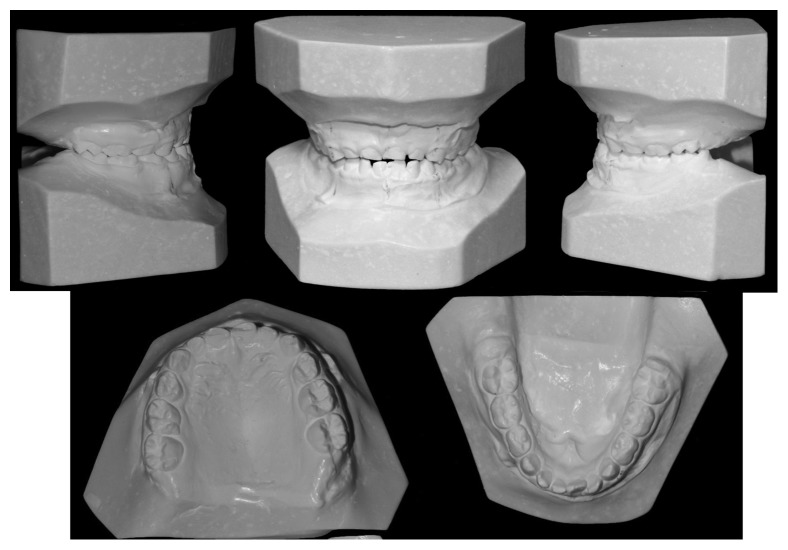
Study casts.

**Figure 6:**
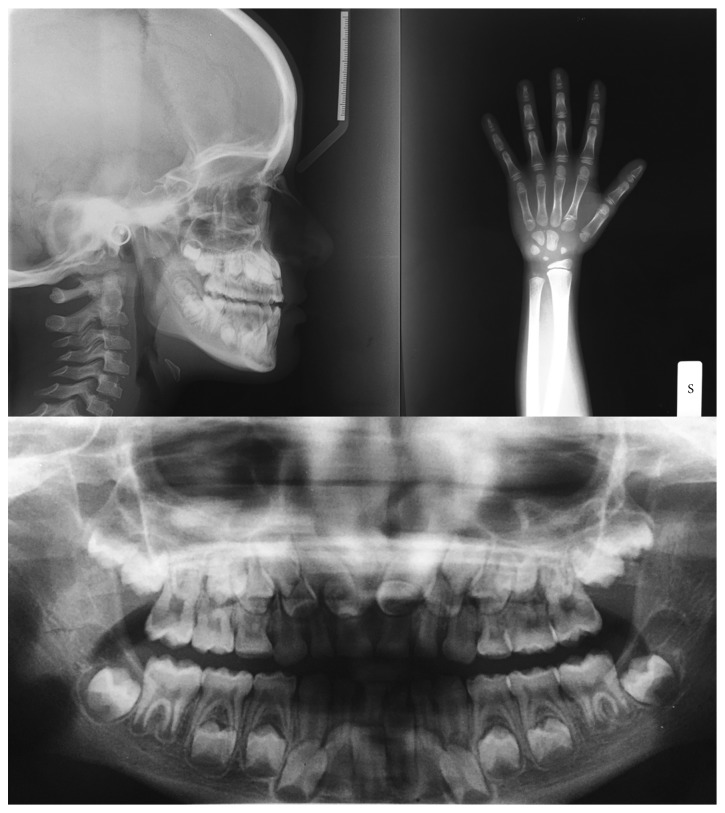
Radiographs of skull and panorex.

**Figure 7:**
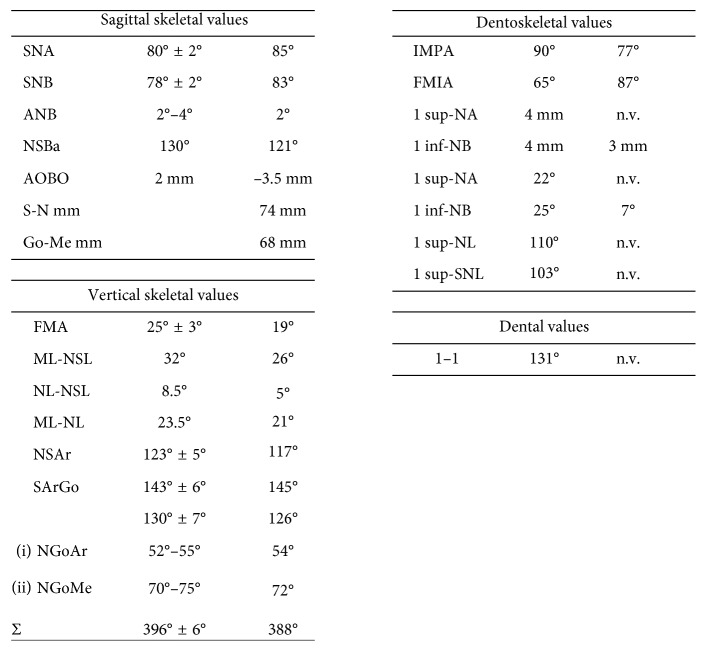
Cephalometric analysis.

**Figure 8:**
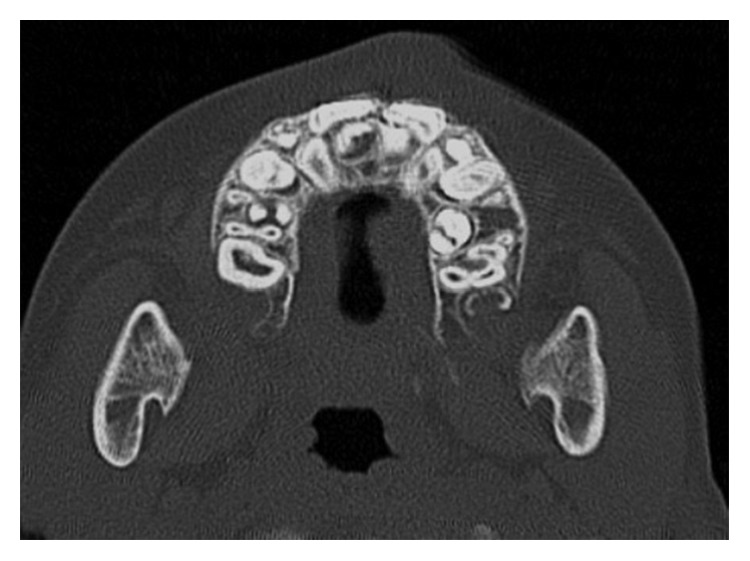
TC dentascan of upper arch.

The orthodontic diagnosis is summarized in Table 11 (Figures 3–8).

#### 4.1.2. Treatment Planning

We will proceed using a two-stage approach, like a “Jerusalem approach” to provide:

- Aesthetic for the patient in the frontal area because the time for eruption of the incisors is passed since several years (although the patients shows delayed eruption of teeth and growth), it's important to exploit the residual *vis a tergo* of these teeth because the canines have the longest and most tortuous path of eruption, and they are located much higher than the crowns of the incisors. To provide this orthodontic approach firstly, the patient will require a skeletal expansion of the upper jaw. A Rapid palatal expander will be designed with bands on 1.6 and 2.6, rests on 5.4 and 6.4, with several arms and loops for the orthodontic traction of the central and lateral upper incisors.

After extraction of deciduous and supernumerary teeth of the anterior upper area, under general anesthesia, the 4 upper permanent incisors will be exposed and immediately bonded for traction under a closed flap. On the lower jaw, he will also need extraction of deciduous teeth. A lingual arch with rests on deciduous first molars and loops for the orthodontic traction of the lower incisors will be employed.

(ii) Only after disinclusion of all incisors, and after surgical removal of deciduous and supernumerary teeth, we will proceed with the lateral sectors, probably using an intraoral double arch, like the arch used with the Delaire mask, but provided of arms for the traction of canines.

#### 4.1.3. Case 2

The patient S.A., older sister of the first patient, came to the orthodontic Department of the University “Sapienza” of Roma in October 2015 at the age of 17, with weight 62 kg and height 1.57 m. Born from caesarian delivery at 37 weeks from mother affected by CCD. She underwent cataract surgery at two years. At the age of 3 years she underwent genetic counseling. X-Ray of the thorax showed missing ossification of median third of right clavicle; normal left clavicle. X-Ray of the skull evidenced Wormian bones. At 5 years of age she underwent adenotonsillectomy. Hypertrophy of turbinates remains. In 2014 another clinical assessment shows overweight (65 kg), short stature (1.52 m), discrete joint laxity, pes planus, genu valgus.

Audiological examination: normal audiometry and impedentiometry with tympanogram type A on the right and type C on the left. The cocleostapedial reflex was present bilaterally for both ipsilateral and contralateral stimulations. No alteration was evident on the TC scan of the middle ear.

*(1) Laboratory findings*: deficit of vitamin D 25-OH (19 ng/ml).

Orthopedic examination in 2015 shows left lumbar scoliosis, dorsal kyphosis, and bilateral scapula humeral anteversion, greater on the right. Bilateral genu valgus recurved. Pes planus valgus, flexible not painful, are also present.

X-ray of the spine (2 projections) and long bones (2 projections) shows convex dorsal left scoliosis with modest accentuation of dorsal kyphosis. Pseudarthrosis of the medial third of right clavicle. Slight hypoplastic left clavicle. Cervicocephalic valgism of both femoral necks. Slight curving of median third of both tibial diaphysis of the tibiofemoral joints.

DEXA *Z*-score −2.8. Skeletal age of 15-16 years.

**Figure 9:**
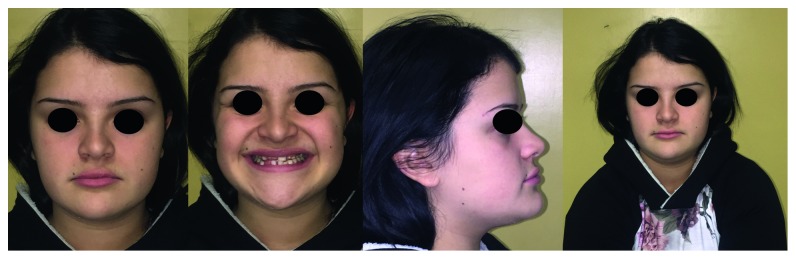
Case 1: pretreatment facial photographs.

**Figure 10:**
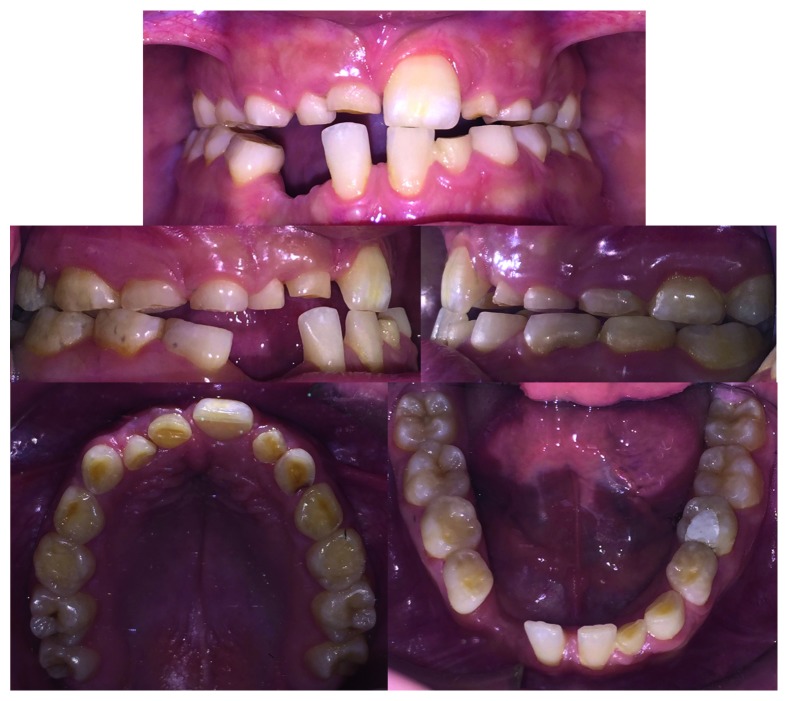
Case 1: intraoral photographs.

**Figure 11:**
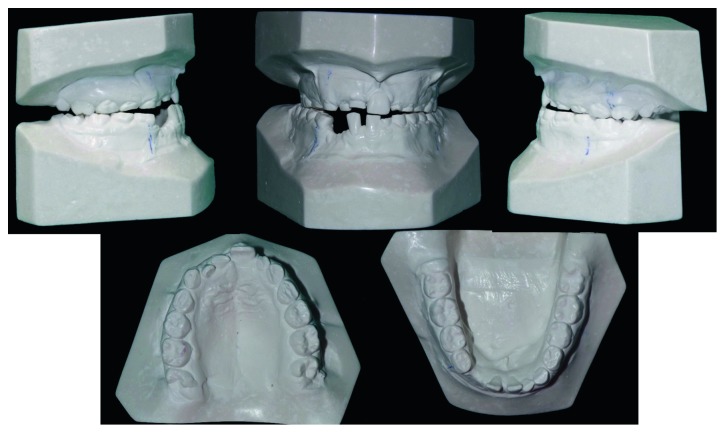
Study casts.

**Figure 12:**
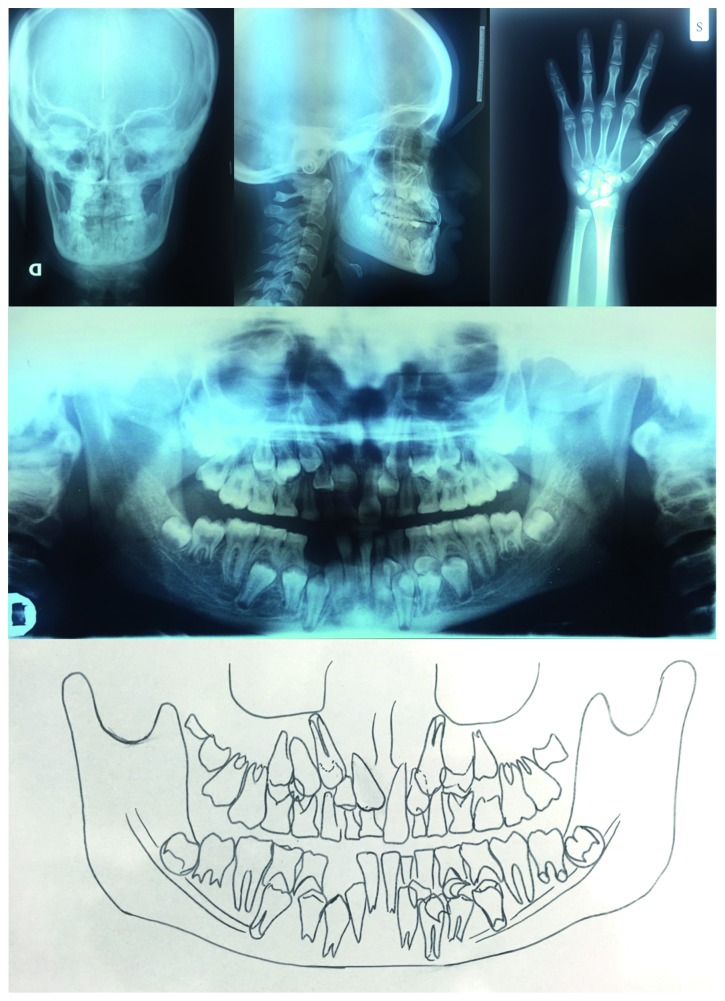
Radiographs of skull and panorex.

**Figure 13:**
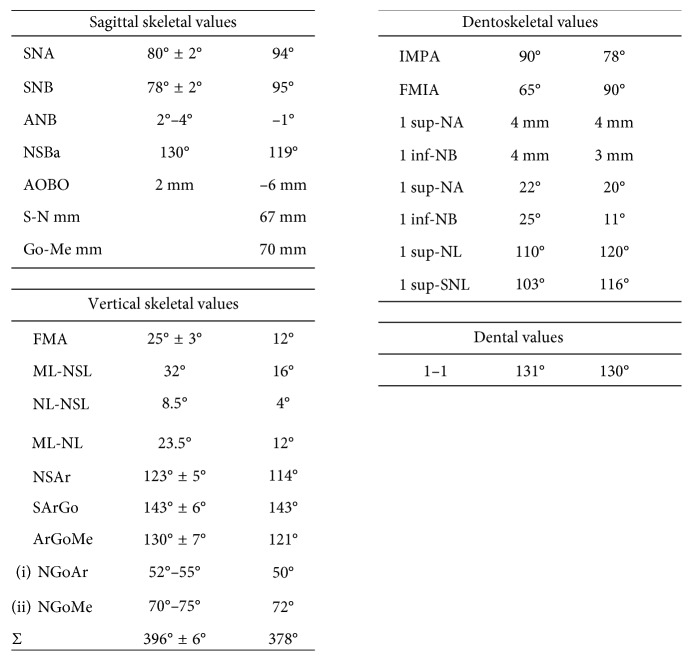
Cephalometric analysis.

**Figure 14:**
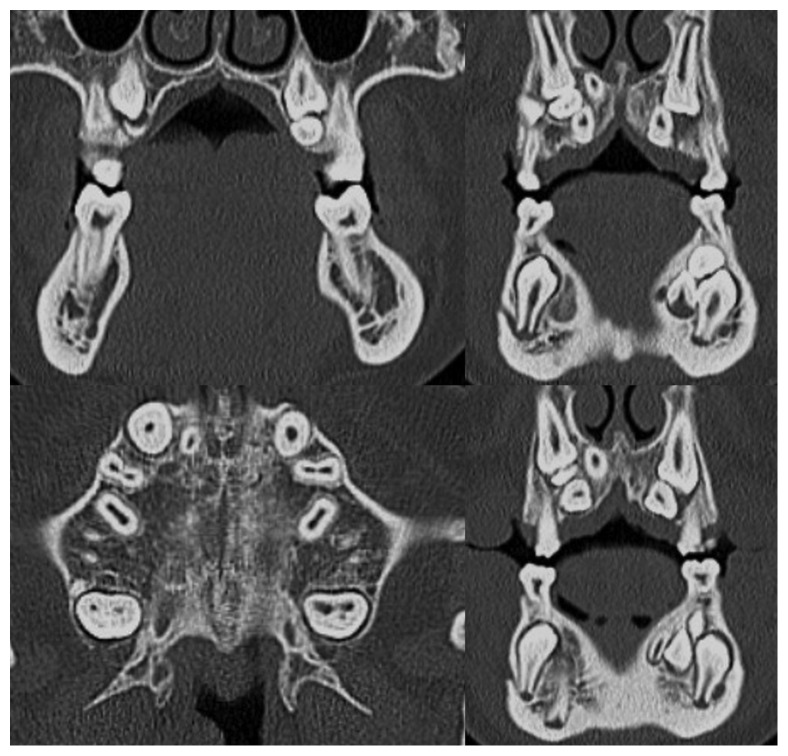
TC dentascan of both arches.

The orthodontic diagnosis is summarized in Table 12 (Figures 9–14).

#### 4.1.4. Treatment Planning

In this case, a single surgical stage approach was chosen, similar to the “Belfast–Hamburg” approach. The treatment will be performed using a double intraoral arch welded on bands on 16 and 26, provided of multiloops on both palatal and vestibular arch and secured with two mini-screws.

“Temporary anchorage devices” (TADs) inserted on the comfort zone of the palate aimed to provide orthodontic traction of 12, 11, and 22 in first place and afterwards canines and bicuspids.

Moreover, we want to use a double intraoral arch for the lower jaw as well. This will be provided of power arms to secure it by means of TADs placed on the vestibular mandibular cortical. All the teeth to be disimpacted will be bonded as possible in the same surgical phase.

#### 4.1.5. Case 3

The mother of the siblings, P.F. 44 years old, weight 86 kg and height 1.54 m is also affected by CCD. She was born in podalic position by vaginal delivery and weighted 2.100 g at birth. She is allergic to Nichel and lanolin alcohol and presents a kidney with double calyx. She underwent surgery 4 times for kidney stones and one time for debridement of temporomandibular joint. When she was 15, she extracted supernumerary teeth and deciduous molars from the III quadrant of the mouth. Until the age of 30 years, she had only lost the four lower incisors and for the rest, was in deciduous dentition. In 2000, she began several surgeries for the extraction of deciduous teeth and supernumerary in the remaining quadrants. She reports to have until 3 supernumeraries for tooth. Finally, she underwent orthognathic surgery for advancement and expansion of the upper maxilla.

In 2014, she is sent by the Department of Maxillofacial Surgery to the Department of “Rare diseases” to assess a complete picture of the clinical and radiological situation before planning a mandibular setback for the resolution of the anterior crossbite. At the CT scan of the head, previously realized, important sclerosis of the cranial vault, with thickening of the interior and exterior planking, was visible: patent sutures and fontanels; important sclerosis of the cranial base, on the petrous part of the temporal bone; poor gasification of antral cavities and mastoids; deviated nasal septum; slight hypertelorism. X-ray of the spine (2 projections) and long bones (2 projections) shows hypoplastic clavicles with pseudoarthrosis of the median third: hypoplastic scapulae and glenoid fossae; small iliac wings; widened sacroiliac synchondrosis with sclerotic processes on the articular surfaces; thickened cortical of the femoral diaphysis; tibiofemoral valgism.

**Figure 15:**
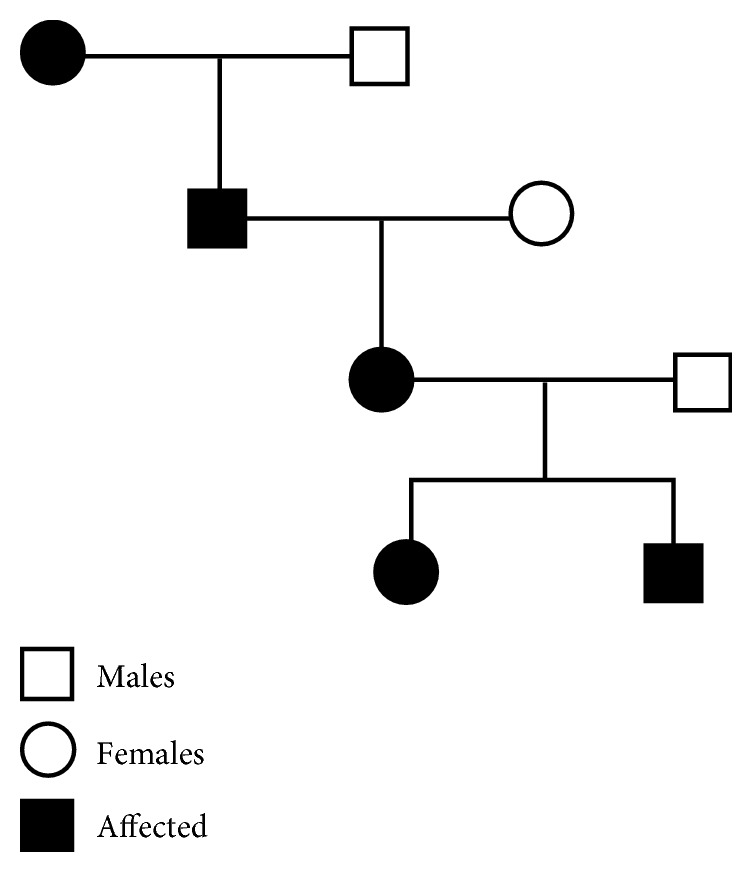
Pedigree of the family affected by CCD.

Laboratory findings show deficit of vitamin D (25(OH)D, 18.1 ng/mL), low levels of phosphates, and elevated level of PTH. The DXA showed a Z-score of −1.4 at the lumbar spine and −0.5 at the left hip. The pedigree of the family shows how the mutation did not skip any generation; this finding strongly points out an autosomal dominant inheritance pattern, characterized by the 50% risk of affected offspring. The mother of the grandfather and grandfather himself were affected by CCD (Figure 15). The patient is not sure yet if she wants to start a new treatment course and refused to realize the lateral Teleradiography because of the numerous previous unsuccessful interventions she underwent and the poor aesthetic results she obtained until today. For this reason, only the clinical, photographic, and plaster model findings were available.

**Figure 16:**
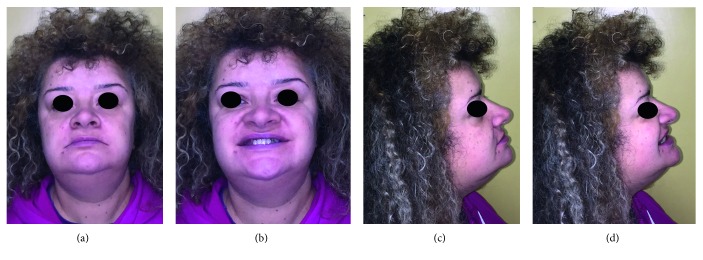
Case 3: extraoral pictures.

**Figure 17:**
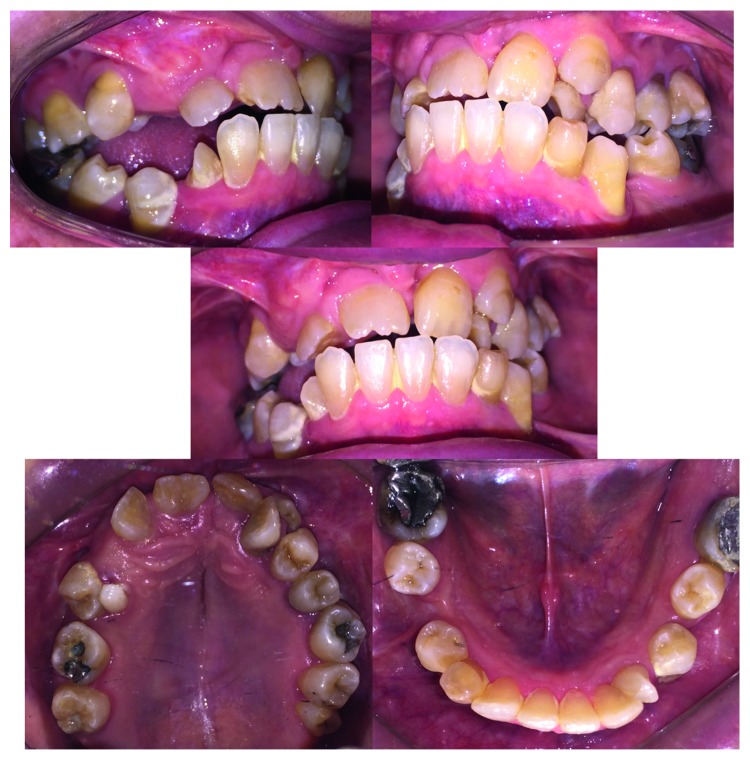
Case 3: intraoral findings.

**Figure 18:**
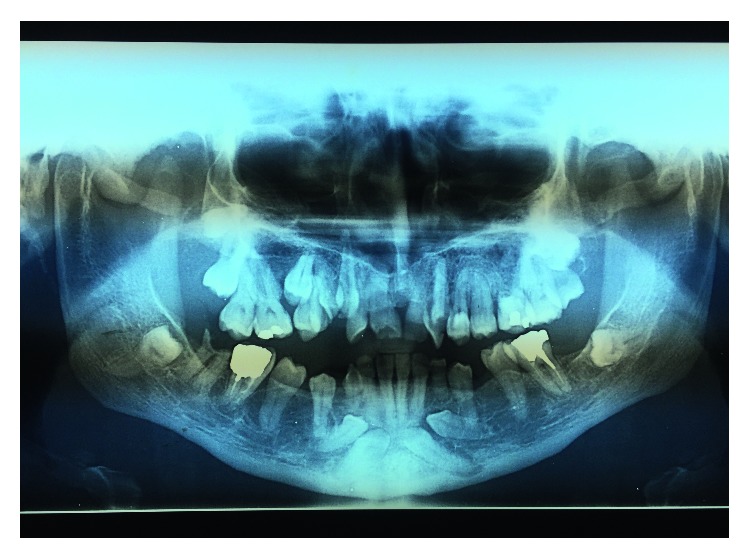
Case 3: radiological findings: OPT.

The orthodontic diagnosis is summarized in Table 13 (Figure 16–18).

#### 4.1.6. Treatment Planning

In this case, the hypnotized treatment planning would be realized with the “Bronx Approach.” A complete engagement of the present teeth in an orthodontic fixed appliance will precede the surgical progressive exposition of the impacted and supernumerary teeth. It is advisable to extract all the supernumerary teeth and of the impacted lower canines, due to their low horizontal position. The alignment and orthodontic traction of the impacted 1.3 will be realized, and the arches will be prepared for a subsequent orthognathic surgery of maxillary advancement and mandibular setback. The extraction of the impacted posterior molars will be realized in that occasion. A complete treatment of the gingivitis will precede all the orthodontic and surgical phases. A restorative reconstruction of the damaged elements will follow the complete orthodontic and surgical procedures.

## 5. Discussion

This report reviews 79 patients with CCD excluding all children under the age of 2 years and over 60. The presence of dental signs such as supernumerary teeth, delayed eruption of permanent teeth, and eruption failure are almost constant in all patients. The family we observed at our Orthodontic Department shows all these dental findings. Such high percentage of dental problems underlines the importance of early diagnosis and multidisciplinary treatment for more reasons.

1. The surgical and orthodontic techniques involved in the dental management of this condition are demanding at the levels of diagnosis, treatment planning, and clinical management. Thus, to achieve the best possible result, the highest degrees of orthodontic and surgical cooperation are necessary.
2. Starting the treatment early with so many surgical procedures can be challenging for a young child but offers the advantage that the patient will have restored function and esthetics in adolescence, which can be psychologically important [9].
3. The natural eruption of teeth with roots should be allowed after the removal of obstacles, such as deciduous or supernumerary teeth.
4. The lack of eruption in patients with CCD is due to failure of resorption of the overlying alveolar bone. When these teeth are uncovered, they show a normal eruption pattern. There can also be mechanical interference from impacted supernumerary teeth.
5. Moreover, sagittal deficiency of the upper jaw was investigated. This characteristic of the skull was found in almost all patients reported.

Some peculiarities are seen in the cephalometric measurements of a patient with CCD. Because the base of the skull is smaller than in patients who do not have the syndrome, measurements such as SNA, SNB, and facial angles appear greatly increased compared with normal ones. In addition, CCD is usually seen in a brachycephalic patient with horizontal growth, expressed by diminished measurements such as the *y*-axis, FMA, and SN/GoGn [9].

The familiar group with cleidocranial dysplasia, studied in the second part of our study, shows most of the characteristics found in the literature.

At the cephalometric examination of our patients, both showed reduced width of the anterior cranial base. The sister has a skeletal class III jaw relationship, according to both ANB and AOBO, which we aim at correcting with Le Fort I advancement of the maxilla at the end of presurgical orthodontic treatment. The mother already underwent a maxillary surgical-assisted expansion, but still requires presurgical orthodontic treatment, extraction of supernumerary teeth, and BSSO surgery for mandibular setback and correction of the anterior crossbite.

The younger son is now in Class I relationship and presents a marked failure to thrive. The mandibular bone growth continues in boys even after completion of statural according to the growth curves of Björk [97].

Sagittal jaw relationship will be monitored during the following years, but the correction of the transversal discrepancy and forced orthodontic eruption of upper and lower incisors are the main concern at this time point.

The reports of literature described various appliances (REP, TPA, and HYRAX) for the transversal correction, and none reported a failure of such procedure.

Finally, it is important to underline the importance of bone anchorage for the orthodontic traction of the impacted teeth. Skeletal anchorage in orthodontics as absolute anchorage provides new opportunity to guide impacted teeth into occlusion and to treat patients with CCD, making it possible to induce eruption simultaneously of the maxillary and mandibular teeth and consequently reducing patients' treatment times and psychological stress [98].

This is our aim in the treatment of the older sister: secure two double-arch wires by fixing them to the median or paramedian anterior palate using two mini-screws in the upper jaw and to the inter-radicular septum of the dentulous alveolar process in the mandible. This will provide maximum anchorage for the forced eruption of impacted teeth, without affecting the only permanent teeth present now (upper and lower first and second molars).

## 6. Conclusion

Revision of the literature is vital for the treatment planning of difficult cases, such as individuals affected by rare diseases, which involve numerous aspects of oral health; CCD is challenging for both the dental team and the patient. The treatment requires a multidisciplinary approach and a strong compliance through many years. A treatment of such condition can last years. It is very important to constantly motivate the patient to full compliance and to keep a scrupulous oral hygiene to avoid the risks that come with orthodontic treatment.

Further studies are required to better understand the cause of delayed eruption of permanent teeth and the abnormal presence of numerous supernumerary teeth that interfere with normal shading of primary dentition.

With the advancement of orthodontic techniques, together with the use of cone-beam for better understanding of the patients' dental situation and the TADs for skeletal anchorage, the treatment results will be greater in future.

According to this review, we chose to treat our patients following a multistep approach thus enhancing the possibilities to achieve the recovery of the most possible number of teeth, to obtain a good occlusion and a better aesthetic, especially of the anterior region. The treatment of the adult patient is obviously requiring a multidisciplinary approach due to the need of surgical correction of the sagittal and transversal discrepancies and due to the need of a prosthetic reconstruction of the missing teeth. A strong suggestion of an early diagnosis and an early treatment seem then to be mandatory to improve the prognosis of the cleidocranial-related anomalies.

## Figures and Tables

**Table 1:** 

Clinical findings	Dentoalveolar characteristics
(i) Open fontanelle or delayed closure of fontanelle (ii) Open sutures (iii) Wormian bones (iv) Skull: usually large broad and brachycephalic type (v) Hypoplasia of maxillae, lacrimal, nasal, and zygomatic bones; discontinuous zygomatic arch; parallel-sided ascending ramus of the mandible; upwards and/or posteriorly pointing coronoid process with less thick masseter muscles than in the age- and sex-matched control subjects (vi) Underdeveloped and narrow paranasal sinuses (vii) Prominent frontal, parietal, and occipital bones (viii) Ocular hypertelorism and a mild exophthalmus (ix) Small and bell-shaped thoracic cage with short, oblique ribs (x) Hypoplastic scapulae with deficiencies in the supraspinatus fossae and acromial facets (xi) Defects of the vertebral column: scoliosis and kyphosis (xii) Pelvis: widened pubis symphysis due to a delay in ossification during adulthood. Hypoplasia and anterior rotation of the iliac wings and wide sacroiliac joints; large femoral epiphyses, broad femoral necks, and frequently coxa vara. Caesarean section of the dysplastic pelvis often necessitates in the pregnant female (xiii) Presence of both proximal and distal epiphyses in the second metacarpals and metatarsals leading to excessive growth and length (xiv) Unusually short bones of the hands and feet, especially the distal phalanges and the middle phalanges of the second and fifth fingers (xv) Cone-shaped epiphyses and premature closure of epiphyseal growth plates lead to shortening of other bones. Tapered appearance to the digit, hypoplastic or dysplastic nails, sometimes absent (xvi) Anomalous muscles secondary to bony involvement	(i) Over retained deciduous teeth without any resorption in roots (ii) Delayed/retarded eruption of permanent teeth because of lessened eruptive potential, although it is not entirely absent (iii) Multiple impacted supernumerary teeth that displace the developing permanent teeth and obstruct their eruption, as a possible result of incomplete or delayed resorption of the dental lamina, which is reactivated at the time of crown completion of the normal permanent dentition (iv) Crypt formation around impacted and ectopic teeth (v) High narrow arched palate and infrequently cleft palate (vi) Partial anodontia (vii) Reduced height of the lower third of the face and a skeletal class III tendency due to the underdevelopment of the maxilla and to an upward and forward mandibular rotation. Markedly reduced vertical development of the alveolar bone, with a shallow buccal and lingual sulcus (viii) Possible nonunion of the symphysis of the mandible (ix) Late but spontaneous eruption of first and usually second permanent molars in both the jaws (x) Delay in the root development of permanent teeth and short and thinner roots (xi) Absence or lack of cellular cementum on the roots of the permanent teeth with no increased thickening of primary acellular cementum, probably due to mechanical resistance to eruption by the dense alveolar bone overlying the unerupted teeth

**Table 2:** Multiple surgery approach (Toronto-Melbourne).

Stage 1	Surgery
5 to 6 years	Extractions/deciduous incisors
9 to 10 years	Extractions/deciduous posterior teeth

Stage 2	Surgery/orthodontics

When first molars have been banded	Surgical exposure of permanent incisors
Following healing from previous surgical exposure	Brackets placed on incisors
9 to 12 years	Surgical exposure of permanent premolars/supernumerary teeth removed
Following healing of previous surgical exposure	Brackets placed on premolars and canines

**Table 3:** Single surgery approach (Belfast-Hamburg).

Intervention	Surgery/orthodontics
	Extractions of all deciduous teeth and all supernumerary teeth
	Exposure of all permanent teeth
	Brackets bonded immediately
	Surgical flap closed
	Orthodontic appliance bonded on fully erupted teeth
	Orthodontic elastic traction of all unerupted teeth

**Table 4:** Two surgery approach (Jerusalem).

Intervention 1	Surgery/orthodontics
Dental age 7 to 8	Extractions/anterior deciduous teeth and all supernumerary teeth
	Exposure of permanent incisors
	Brackets bonded immediately
	Surgical flap closed

Intervention 2	Surgery/orthodontics

Dental age 10 to 11	Extractions/remaining deciduous teeth
	Exposure of unerupted premolars and canines
	Brackets bonded immediately
	Surgical flaps closed

**Table 5:** Surgical-prosthetic approach (Bronx).

Surgery	Surgery
Intervention 1	Extractions/deciduous teeth and all supernumerary teeth
	Surgical flaps closed

Intervention 2	Exposure of unerupted teeth
	Brackets bounded immediately
	Surgical flaps closed

Intervention 3	Le Fort I Osteotomy. Orthognatic surgery
	Placement of dental implants

**Table 6:** 

	Reference	Rationale for exclusion
1	Anthonappa et al. [17]	No dental findings, genetic analysis
2	Takenouchi et al. [18]	No dental findings, patient with cognitive decline
3	Rallan et al. [19]	Nonsyndromic case
4	Wang and Neustein [20]	No dental findings
5	Matsushita et al. [21]	Age of case report (2-year-old boy)
6	Broeks et al. [22]	Age of case report (infant)
7	Gardham et al. [23]	Age of case report (infant)
8	Northup at al. [24]	Age of case report (infant)
9	Cardoso et al. [25]	Age of case report (infant)
10	Shen et al. [26]	No dental findings
11	Manjunath et al. [27]	No dental findings
12	Pamuk et al. [28]	No dental findings
13	Issever et al. [29]	No dental findings
14	Pal et al. [30]	No dental findings
15	Fernandes et al. [31]	No dental findings
16	Cunningham et al. [32]	Age of case report (infant)
17	Campos Junior et al. [33]	Age of case report (infant)
18	Izumi et al. [34]	Age of case report (infant)
19	Fernandez et al. [35]	No dental findings
20	Goto et al. [36]	Age of case report (infant)
21	Patel and Athavale [37]	Age of case report (infant)
22	Golan et al. [38, 39]	same case already reported
23	Sakai et al. [40]	Age of case report (75-year-old man)

**Table 7:** 

Authors (date)	Cases (*n*)	Sex m f	Mean age	Supernumerary teeth	Eruption failure	Delayed eruption of permanent teeth	Hypoplastic maxilla	Maxillary contraction and related therapy	Clavicular sign	Miscellaneous comments
Madeira et al. (2015) [41]	1	1	21	Yes	Yes	Yes	Yes	Yes	Yes	Le Fort I osteotomy: 5 mm maxillary advancement; BSSO mandible advancement of 2.4 mm with counterclockwise occlusion
Zhang et al. (2015) [42]	3	2 1	14	Yes	Yes	Yes	Yes	Yes	Yes	
Guo et al. (2015) [16]	1	1	52	Yes	Yes	n.a.	Yes	n.a.	Yes	Sporadic, depressed frontal area, hypertelorism
Paul et al. (2015) [43]	2	2	15,5	Yes	Yes	Yes	Yes	Yes, class III malocclusion	Yes	Hypertelorism and frontal bossing
Lu et al. (2015) [44]	1	1	20	Yes	Yes	Yes	Yes	Yes, bilateral anterior and posterior crossbite, class III malocclusion, high-arched palate	Yes	Frontal bossing, ahypoplastic midface with hypertelorism, a depressed nasal bridge, *fusion of the primary teeth*
Rocha et al. (2014) [45]	1	1	22	Yes	Yes	Yes	Yes	Yes (crisscross elastics)	Yes	
Chen et al. (2014) [46]	1	1	29	Yes	Yes	Yes	Yes	Yes	Yes	Brachydactyly, joint laxity, incomplete gasi cation of the frontal sinus
Hardy et al. (2014) [47]	1	1	31	Yes	n.a.	n.a.	n.a.	n.a.	Yes	Acute, traumatic, posterior *dislocation of both glenohumeral joints* in a case of bilateral clavicular agenesis
Gömleksiz et al. (2014) [48]	1	1	24	Yes	Yes	Yes	Yes	submucosal cleft palate causing rhinolalia	Yes	Hearing loss; dyspnea, fatigue, hypertelorism, frontal bossing
Bedeschi et al. (2014) [49]	1	1	13	n.a.	Yes	Yes	Yes	Yes	Yes	Hypertelorism, bilateral flat foot
Park et al. (2013) [14]	2	1 1	13	Yes	Yes	Yes	Yes (class III; Le Fort I both of them)	Yes (TPA), 9 mm anterior crossbite hyrax expander	n.a.	Case 1 (12-year-old boy): broad forehead, hypertelorism. Case 2 (14-year-old girl): bilateral TMJ clicking, Agenesia 37
Back and Pollock (2013) [50]	1	1	17	n.a.	Yes	Yes	Yes	Yes, skeletal class III	Yes	Flattening of the frontal bones, hypertelorism
Vij et al. (2013) [51]	1	1	15	Yes	Yes	Yes	Yes	Yes	Yes	Open skull sutures, obliterated maxillary sinus
Callea et al. (2012) [52]	1	1	11	No, absence of 3.2	Yes	Yes	n.a.	Yes, REP	Yes	Mild bilateral sensorineural hearing loss, mandibular hypoplaia, brachycephaly, frontal bossing, large fontanelles
Nel et al. (2012) [53]	1	1	10,5	Yes	Yes	Yes	No, class II	n.a.	Yes	
Sberna et al. (2012) [54]	2	1 1	13,5	Yes	Yes	Yes	Yes	Yes	Yes	Anomalies of roots, altered morphology of lateral incisors, ectopic canines
Mortellaro et al. (2012) [55]	2	2	24	Yes	Yes	Yes	Yes	Yes	Yes	Case 1: a 15-year-old boy; case 2: a 12-year-old girl
Fang et al. (2011) [56]	1	1	18	Yes	Yes	Yes	Yes	Yes	Yes	Patent fontanelles, wide cranial sutures, protruding mandible
Petropoulos et al. (2011) [57]	1	1	45	Yes	Yes	Yes	Yes	Yes	Yes	
Berg et al. (2011) [15]	2	1 1	13	Yes	Yes	Yes	Yes	Yes, expansion and SS magnetic keepers laser-welded onto the transpalatal arch (TPA)	Yes	Female patients required also Le Fort I surgery, genioplasty, and insertion of 2 implants in the premolar areas bilaterally
Mehta et al. (2011) [58]	2	2	12.5	Yes	Yes	Yes	Yes	Yes	Yes	Hypertelorism, depressed nasal bridge, frontal prominence
Trigui et al. (2011) [59]	2	1 1	3	n.a.	n.a.	n.a.	Yes	Yes	Yes	Hypertelorism, bilateral flat foot coxa vara, frontal bossing, wormian bones
Dalessandri et al. (2011) [60]	1	1	15	Yes	Yes	Yes	Yes	Yes, bilateral crossbite	Yes	
Karagüzel et al. (2010) [61]	1	1	3.5	Yes	n.a.	n.a.	Yes	Yes	Yes	Mandibular retrognathism, brachiocephalic head
Kamatham et al. (2011) [62]	1	1	11	Yes	Yes	Yes	Yes	n.a.	No	
Kamamoto et al. (2010) [63]	1	1	28	Yes	Yes	Yes	Yes	Yes	Yes	
Xuan et al. (2010) [64]	2	1 1	25.5	Yes	Yes	Yes	Yes	Yes	Yes	Parents not affected by CCD, novel nonsense mutation (p.E366X) was identified
Mohan et al. (2010) [65]	1	1	9	Yes	Yes	Yes	Yes	Yes	Yes	Exertional dyspnea, repeated ear infections and sinusitis, prominent forehead, with hypertelorism, a depressed nasal bridge
Wang et al. (2010) [66]	3	2 1	11.83	Yes	Yes	Yes	n.a.	n.a.	Yes	Father, son, and daughter
El-Gharbawy et al. (2010) [67]	1	1	6	Yes	Yes	Yes	Yes	Yes	Yes	Kyphoscoliosis, frenuloplasty for ankyloglossia
Dhanpal et al. (2009) [68]	1	1	15	Yes	Yes	Yes	Yes	Yes	Yes	Two fused roots of the maxillary primary canines
Ioscovich et al. (2009) [69]	1	1	23	n.a.	Yes	Yes	Yes	Yes	Yes	Hypertelorism, frontal bossing, open fontanels
Chelvan et al. (2009) [70]	1	1	10	Yes	Yes	Yes	Yes, class III incisor relationship	yes: mild bilateral crossbite	Yes	Frontoparietal bossing, prominent orbital ridges, mild hypertelorism
Suresh (2009) [71]	1	1	38	Yes	Yes	Yes	Yes	Yes	Yes	
Kang et al. (2009) [72]	1	1	19	n.a.	Yes	Yes	Yes	Yes, orthognatic class III correction	Yes	Hyperhidrosis of the palms and soles, stuffy phalanges, hypoplastic toe nails
Farronato et al. (2009) [73]	1	1	28	Yes	Yes	Yes	Yes	Anterior crossbite, class III	Yes	Frontal bossing, depressed suborbital region, defective nasal bones
Rasool et al. (2008) [74]	1	1	12	Yes	Yes	Yes	Yes	n.a.	Yes	
Gonzalez et al. (2008) [75]	1	1	7	n.a.	n.a.	Yes	Yes	Yes, high-arch palate	Yes	Hypertelorism, conductive hearing loss
Purandare et al. (2008) [76]	1	1	17	Yes	n.a.	n.a.	n.a.	n.a.	Yes	Abnormal dentition, short stature, scoliosis, joint laxity, recurrent respiratory infections, learning disability
Hemalatha and Balasubramaniam (2008) [77]	1	1	12	Yes	Yes	Yes	n.a.	n.a.	Yes	Tongue-thrusting habit; a Nance palatal arch space maintainer with a fixed tongue crib
McBrien et al. (2008) [78]	1	1	18	n.a.	n.a.	Yes	n.a.	Yes	Yes	
Tang et al. (2007) [79]	1	1	34	n.a.	Yes	Yes	Yes, prominent lower jaw	n.a.	Yes	Hyperplastic nails
Kobayashi et al. (2007) [80]	1	1	27	Yes	Yes	Yes	Yes	n.a.	Yes	Shortened middle phalanges of the index and little fingers; anterior subluxation of the atlantoaxial joint causing myelopathy
Kuroda et al. (2007) [81]	1	1	10,11	Yes	Yes	Yes	Yes, protrusive chin	n.a.	n.a.	
Tanaka et al. (2006) [82]	4	2 2	22.65	Yes	Yes	Yes	Yes	Yes	Yes	Family: mother 47.7 y, son 16.3 y, son 14.1 y, daughter 12.5 y
Olszewska (2006) [83]	1	1	40	Yes	Yes	Yes	Yes, reverse OJ in the area of incisors	n.a.	Yes	Suffered from recurrent infections of sinuses, upper airways and ears
Mohan et al. (2006) [84]	1	1	22	Yes	Yes	Yes	Yes, pseudoprognatic	Yes	No	Parallel ascending rami, short clavicle, and hypoplsia
Tokuc et al. (2006) [85]	1	1	22	Yes	Yes	Yes	n.a.	n.a.	Yes	Open anterior fontanel, woman was using a prosthesis
Suba et al. (2005) [86]	1	1	13	Yes	Yes	Yes	Yes	Yes, severe crossbite therapy: (1) removable appliance to expand the maxilla, (2) multiband treatment of the maxillary dental arch, (3) transversal expansion of the narrow mandibular arch by a Y-shaped screwed appliance, (4) Delaire mask	Yes	Narrow ascending mandibular rami with parallel-sided anterior and posterior borders; open fontanelles and hypoplastic nasal bone; NSBa ML-NL angles narrower, incisal angle wider than normal
Vakili and Jalali (2005) [87]	1	1	17	Yes	n.a.	n.a.	n.a.	n.a.	Yes	Hypogonadotropic hypogonadism with delayed puberty
Angle and Rebellato (2005) [88]	1	1	10,1	Yes	Yes	Yes	Yes, skeletal class III	Yes, posterior crossbite	Yes	Frontal bossing, left thoracic scoliosis
González López et al. (2004) [89]	3	3	24.3	Yes	Yes	Yes	Yes	Yes, high palate and anterior crossbite	Yes	Moderate bilateral hypoacusia in the mother; hypertelorism in the daughter
Furuuchi et al. (2005) [90]	1	1	21	Yes	Yes	Yes	n.a.	n.a.	Yes	Parallel ascending rami, hypoplastic zygomatic bone, and discontinuous zygomatic arch
Cogulu et al. (2004) [91]	1	1	2	n.a.	Yes	n.a.	Yes	Yes	Yes	Microstoria, clinodactyly, brachydachtyly, nail hypoplasia, horse-shoe kidney, hypospadias, undescended testis, coxa vara, pes planes, genau valga
Petropoulus et al. (2004) [92]	1	1	42	Yes	Yes	Yes	Yes	Yes	Yes	
Yildirim et al. (2004) [93]	1	1	18	n.a.	n.a.	Yes	Yes	Yes	Yes	Enamel hypoplasia, narrow thorax
Unger et al. (2002) [94]	1	1	11.2	Yes	Yes	Yes	n.a.	n.a.	Yes	Hypertelorism, upslanting palpebral fissures
Morava et al. (2002) [95]	2	2	21	No	Yes	Yes	n.a.	n.a.	Yes	Metabolic signs of hypophosphatasia, i.e., low ALP and an increase in P5P and phosphoethanolaminuria
Golan et al. (2002) [38, 39]	1	1	18	Yes	Yes	Yes	Yes	molar crossbite	Yes	Widened thumbs and first toes, iris cyst in the right eye
Machuca-Tzili et al. (2002) [96]	2	1 1	10	n.a.	n.a.	Yes	Yes	Yes	Yes	Case 1 (4 y): conductive deafness (adenoidectomy and septoplasty), anterior cataract on the right eye
										Case 2 (16 y): frontal and parietal bossing, hypertelorism, conductive deafness, clinodactyly of the V finger in both hands

**Table 8:** 

	Number of patients	(%)
Total of patients	79	100
Females	46	58.2
Males	33	41.8

**Table 9:** 

Clinical findings		
	Females	Males
Mean age	18.85 years	18 years
Supernumerary teeth	100%	100%
Eruption failure	100%	100%
Delayed eruption of permanent teeth	100%	100%
Maxillary contraction	100%	100%
Clavicular sign	100%	100%
Hypoplastic maxilla	98%	94%
Missing of permanent teeth	2.5% (two cases)	0%
Class II malocclusion		1.2% (one case)
Class III malocclusion		98.8%

**Table 10:** 

Treatment options		
	Females	Males
REP	100%	100%
Hyrax	100%	100%
Transpalatal arch (TPA)	100%	100%
Removable appliance (Schwartz appliance)	100%	100%
Clavicular sign	100%	100%

**Table 11:** Case 1: orthodontic diagnosis summary.

Facial and functional findings	Dental findings	Skeletal findings
Brachyfacial	Mixed dentition: All deciduous teeth except 3.1 and 1.6, 2.6, 3.6 and 4.6	U-shaped upper and lower arches
Concave profile	5 supernumerary teeth in the upper jaw	Class I skeletal relationship
Normotonic facial muscles	Class I molar relation-ship, class I canine deciduous relationship	Reduced width of anterior cranial base
Mouth breathing	Median lines are not coincident, the inferior is deviated on the right	Reduced total divergency
Atypical swallowing	Overjet reduced	Anterior rotation of maxilla
Normal insertion of labial and lingual frenulum	Overbite reduced	Counterclockwise rotational growth
		Endoinclined lower incisors

**Table 12:** Case 2: orthodontic diagnosis summary.

Facial and functional findings	Dental findings	Skeletal findings
Brachycephalic	Mixed dentition with presence of 16, 17, 21, 26, 27, 31, 36, 37, 41, 46, 47	Contraction of upper jaw
Concave profile with prognathic chin	6 supernumerary teeth in the maxilla and 5 in the lower jaw	Class III skeletal relationship
Normotonic facial muscles and masticatory muscles	Class III molar relationship bilaterally	Reduced width of anterior cranial base
Nasal breathing	Class III deciduous canine relationship on the left (lower canine is missing on the right)	Reduced divergence (SN/ML) 16°
Normal swallowing	Centered dental midlines	Anterior rotation of maxilla
Normal insertion of labial and lingual frenulum	Overjet reduced	Counterclockwise rotation of maxilla and mandible
	Overbite reduced	Endoinclined lower incisors, proclined upper incisors with normal interincisor angle

**Table 13:** Case 3: orthodontic diagnosis summary.

Facial and functional findings	Dental findings
Brachycephalic, symmetric	Presence of 1.1, 1.2, (1.3 impacted) supernumerary, 1.4, 1.6, 1.7, (1.8 impacted) 2.1, 2.2, 2.3, 2.4, 2.5, 2.6, 2.7, 2.8, (2.9 impacted) 3.1, 3.2, 7.3 (3.3 impacted) 3.4, 3.5, (supernumerary impacted) 3.6, (3.8 impacted), 4.1, 4.2, 4.3 (2 impacted supernumerary) 4.4, 4.5, 4.6, (impacted 4.8).
Concave profile with prognathic chin	6 supernumerary teeth in the maxilla and 5 in the lower jaw
Reverse smile arch, with lower incisors exposure	Class III molar relationship bilaterally
Not visible keratinized gengiva	Class III canine relationship nonapplicable
Normotonic facial muscles and masticatory muscles	Not centered dental midlines
Nasal breathing	Overjet: −0.5 mm; overbite: 0 mm
Normal swallowing	Accentuated Spee curve
	Crossbite between 16 and 46
	Dental midlines noncoincident (upper deviated on the left)
	Poor oral hygiene
	Endodontic treatment on 3.6 and 4.6
	Mobility of 1.6, 2.6, 3.6, 4.6
